# Maryland

**Published:** 1895-06

**Authors:** A. W. Clement

**Affiliations:** Secretary


### Maryland.

The Veterinary Medical Examining Board of the State of Maryland, while they have not what might be termed a satisfactory law, are still working hard and feel confident that with the aid of those practitioners now registered the next legislature may be influenced so to amend the present law as, at any rate, to approximate a satisfactory condition of things. There are now registered some one hundred and fifty-four names, of which number forty-four are graduates, and one hundred and forty-one new graduates, with one exception, men who have been in practice for years prior to the passing of the act. One man, who had been in practice but three years, took advantage of the act and passed a satisfactory examination before the board. He was at the time a student at a veterinary college. It is possible that some of the men registered as existing practitioners may have stretched their consciences a little, but all registrations are accompanied by sworn affidavits, so that at any time that it may be proven that such men had not been in practice for such length of time, they can be prosecuted for perjury and their names erased from the roll of registered practitioners. There have been no prosecutions made yet to test the validity of the law, but the board contemplates such a procedure in the near future. The law has been followed to the letter, and no names have been registered as non-graduates since January, 1895. The examination for graduates of schools requiring a three-years'

course of study will be held the last of this month or the first part of July. No applications will be received from graduates of schools requiring less than three years, unless they show evidence of having graduated prior to January, 1895, in which case they may be registered, even without passing any examination. The board is somewhat embarrassed now from lack of funds, but it is to be hoped that this will be overcome when those registered have been called together in session, and the result of the work of the board has been laid before them. The fact that so many men have been registered, and that the State is so well represented, geographically, in the registration, together with the great interest taken in the law by those who are registered, gives the board good ground for believing that they will be successful in their efforts before the next Legislature to have the law greatly improved. All those who honorably believe in making the profession better must certainly wish us success. There is a disposition among a few of our graduates to oppose the law, as favoring the ungraduate practitioner too much, but the framers of the law considered that they could not be too liberal at first, and the experience of the board has proven the wisdom of the thought, for upon the ungraduated registered practitioners must depend further progress in legislation.

A. W. Clement,
Secretary.

